# A Real-Time Measurement System for Long-Life Flood Monitoring and Warning Applications

**DOI:** 10.3390/s120404213

**Published:** 2012-03-28

**Authors:** Rafael Marin-Perez, Javier García-Pintado, Antonio Skarmeta Gómez

**Affiliations:** 1 Department of Information and Communication Engineering, University of Murcia, Campus de Espinardo, E-30100, Murcia, Spain; E-Mail: skarmeta@um.es; 2 Euromediterranean Water Institute, Campus de Espinardo, E-30100, Murcia, Spain; E-Mail: jgarciapintado@gmail.com; 3 National Centre for Earth Observation, University of Reading, Harry Pitt Building, 3 Earley Gate, Whiteknights, Reading RG6 6AL, UK

**Keywords:** real-time data acquisition, sensor network, hydrological monitoring, flood warning system

## Abstract

A flood warning system incorporates telemetered rainfall and flow/water level data measured at various locations in the catchment area. Real-time accurate data collection is required for this use, and sensor networks improve the system capabilities. However, existing sensor nodes struggle to satisfy the hydrological requirements in terms of autonomy, sensor hardware compatibility, reliability and long-range communication. We describe the design and development of a real-time measurement system for flood monitoring, and its deployment in a flash-flood prone 650 km^2^ semiarid watershed in Southern Spain. A developed low-power and long-range communication device, so-called DatalogV1, provides automatic data gathering and reliable transmission. DatalogV1 incorporates self-monitoring for adapting measurement schedules for consumption management and to capture events of interest. Two tests are used to assess the success of the development. The results show an autonomous and robust monitoring system for long-term collection of water level data in many sparse locations during flood events.

## 1. Introduction

A warmer climate, with its increased climate variability, will increase the risk of both floods and droughts [1], whose management and mitigation are important to protect property, life, and natural environment. Real-time accurate monitoring of hydrologic variables is key for flood forecasting, as well as for optimizing related warning systems for damage mitigation. Recent studies show that in the specific case of semiarid and arid areas, adequate deployment of monitoring networks is essential to a real understanding of the underlying processes generating run-off in storm events, and to achieve effective emergency systems (e.g., [2]).

Traditionally, researchers have directly collected data at the places of interest. This has now been commonly substituted by automatic sensor and datalogger systems, which provide high temporal data resolution, while reducing operational human resource requirements. Dataloggers permit local automatic and unattended data gathering, and reduce environmental perturbation. However, data retrieval from standard dataloggers and storage in processing and control/warning centers still has to be done either manually, which prevents its applicability in flood warning systems, or through wired connections, which leads to substantial investments and operational costs. To confront these problems, sensor network technology has been proposed in many monitoring applications [3]. Yet, specific literature on sensor network for flood forecasting is sparse, with only a few examples available (e.g., [4–8]).

Basically, a sensor network comprises a set of nodes, where each node includes a processor, a wireless radio module, a power supply, and is equipped with sensor hardware to capture environmental data. Each node performs the tasks of data gathering, physical parameter processing, and wireless data transmission to the control server. Specifically, for hydrologic applications, sensor nodes must also fulfill a number of additional requirements:

- Power lifetime: Power sources are often not available at the locations of hydrological interest. Moreover, these locations are usually unprotected, and if renewable energy devices are used, there are prone to vandalism or theft. Thus, sensor nodes must have low-consumption, which along with existing standard batteries, should last at least one hydrologic cycle.
- Sensor hardware compatibility: Most hydrologic sensor nodes include a datalogger device connected through a cable to one or more measurement instruments. The datalogger must provide multiple wired interfaces to be able to communicate with a range of specific sensor hardware interfaces. This also involves issues of power supply, and selective time for power dispatching, which leads to optimal power management and facilitates the expansion of connected instruments.
- Reliability: Harsh weather conditions may cause failures in the wireless communication over the monitoring network. Backup mechanisms in local sensor dataloggers must be used to avoid information losses in unexpected crashes.
- Long-range communication: Hydrologic measurement locations are commonly sparse over large areas, and far away from the control center (*i.e.*, tens or hundreds of kilometers). Sensor nodes must have a peer-to-peer connection with the control center.

In general, these, sometimes opposing, requirements are difficult to be satisfied by existing developed solutions. For example, multiple sensor readings and long-range communication are high power-consumption tasks, which diminish battery lifetime. For instance, many existing wireless solutions for agriculture applications (e.g., [9–11]) use a set of tens or hundreds of motes, which collaborate to gather dense data in a small area. Motes have low consumption, but they provide limited sensor interfaces, and short-range communication. On the other hand, several hydrologic and meteorologic applications have been implemented with a few wireless datalogger stations, which individually obtain multi-sensor data in a few sparse locations over a large area (e.g., [5,12–14]). These dataloggers permit high computing and long-range communication. However, they have an excessive investment cost and a high consumption that may be, in the long-term, unsustainable.

This paper describes the design, development, and deployment of a real-time monitoring system for hydrological applications. The paper is focused on the description in detail of our wireless datalogger device, so-called DatalogV1 [15], which combines the low consumption of motes and the reliable communication of most powerful multi-sensor datalogger stations in order to satisfy the requirements of flood warning system scenarios. The DatalogV1 provides automatic monitoring and long-term autonomy in sparse points over large areas.

To demonstrate the goodness of the DatalogV1 design, we deployed a monitoring network in the *Rambla del Albujón* watershed, in Southern Spain. The severity of flash floods in the *Rambla del Albujón* has caused important environmental and economic damages over the last years. Accordingly, the wireless monitoring network is intended to provide real-time accurate hydrologic information to support an operational model-based flood warning system. This is an excellent test to asses the DatalogV1 performance and success in a real case scenario.

The remainder of the paper is organized as follows. Section 2 introduces the context of environmental monitoring and flood warning systems. Section 3 depicts our hydrologic monitoring scenario. Section 4 presents the design of DatalogV1 hardware. Section 5 shows the implementation of DatalogV1 software. Section 6 describes the architecture developed for remote hydrologic monitoring. Section 7 describes the deployment of the monitoring network in the *Rambla del Albujón* watershed. Section 8 shows the results obtained regarding power consumption and data collection. Section 9 provides concluding remarks.

## 2. Environmental Monitoring

Environmental monitoring is the most popular application for sensor networks. At present, sensor networks have been applied for a number of applications as, e.g., soil moisture monitoring [16], solar radiation mapping [17], aquatic monitoring [18], glacial control and climate change [19], forest fire alarm [20], landscape flooding alarm [21], and forecasting in rivers [22]. The ability to place autonomous and low cost nodes in large harsh environments without communication infrastructure enables accurate data collection directly observed from interest areas. With sensor networks, environmental data can be observed and collected in real-time, and used for forecasting upcoming phenomena and sending prompt warnings if required.

### 2.1. Model-Based Flood Warning System Context

The developed sensor network was incorporated within the context of a model-based flood warning system in the *Rambla del Albujón* watershed. A model-based flood warning system, for mitigating the effects of flooding on life and property, incorporates a catchment model based on observed/forecasted rainfall and telemetered observations of hydrologic state variables at various locations within the catchment area. Generally, observed variables are flow and/or water level in channels. Also, other variables such as soil moisture and piezometric levels may be of interest, depending on the watershed response. Real-time updating of the flood forecasting involves the continual adaptation of the model state variables, outputs and parameters, so that the forecasts for various times into the future are based on the latest available information and are optimized, in some sense, to minimize the forecasting errors (e.g., [23]). This is the process of *data assimilation*. Implementation of environmental sensor networks for data assimilation within model-based flood warning systems involves complex engineering and system challenges. These systems must withstand the event of interest in real-time, remain functional over long time periods when no events occur, cover large geographical regions of interest to the event, and support the variety of sensor types needed to detect the phenomenon [8].

## 3. Hydrological Monitoring and Forecasting in the Rambla del Albujón Watershed

**Figure 1.**
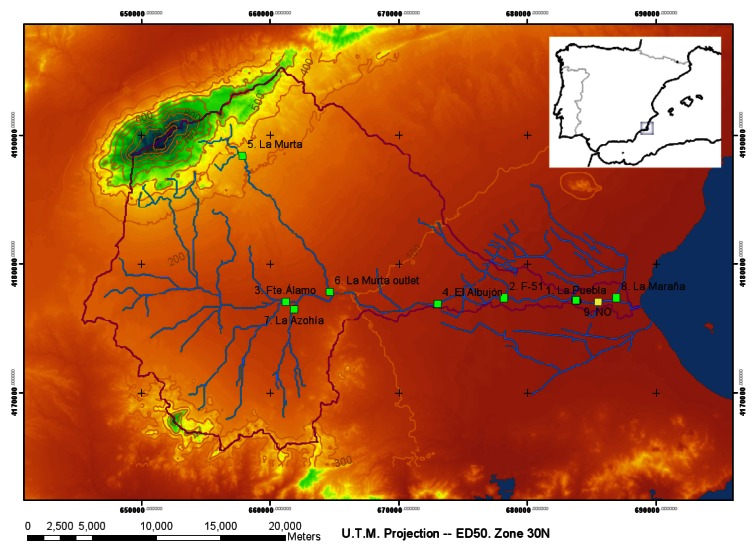
Deployment scenario. The embedded image shows the location of the *Rambla del Albujón* watershed at the Southeast of the Iberian Peninsula. The violet line describes the watershed boundary drawn on a digital terrain model (DTM). Within the watershed, the main channel network is shown in blue, and labeled squares indicate deployed gauge locations.

The *Rambla del Albujón* watershed (650 km^2^) is the main drainage catchment in the *Campo de Cartagena* basin, in Southern Spain (see Figure 1). The main channel in the watershed is 40 km long and flows into the *Mar Menor*; one of the big coastal lagoons in the Mediterranean (135 km^2^). The *Campo de Cartagena* basin is an area with semiarid Mediterranean climate, where the average temperature ranges from 14 °C to 17 °C, mean potential evapotranspiration is 890 mm yr^−1^ and mean precipitation is 350 mm yr^−1^. Most rainfall comes in short-time storm events, and the watershed hydrologic response is highly complex and non-uniform.

Previous studies have shown the complex flash-flood response of the *Rambla del Albujón* watershed and the importance of spatially distributed observation for adequate forecasting (e.g., [2]). Also, for flooding evaluations, stage gauges provide an advantage over flow gauges that the observations remain unbiased when flow goes out of banks, in which case the validness of calibrated rating curves (stage-flow relationships) is prevented. In this sense, remotely-sensed information (from aerial photography and/or satellites) is appealing as it contains much more spatial information than typical stage gauge networks in operational watersheds. Accordingly, recent studies are evaluating the potential of aerial photography and remotely sensed (from satellites) synthetic aperture radar to provide measurements over large areas of water levels and flood extents in lakes and rivers (e.g., TerraSAR-X or COSMO-Skymed constellations [24]). However, the current low temporal frequency of satellite acquisitions relative to gauging station sampling indicates that remote sensing still does not represent a viable replacement strategy for data assimilation into model-based forecasts [25]. Also, before the flow goes out of banks, the accuracy of standard stage gauges is higher than that provided by airborne information, which is key for early warnings. Thus, if economically viable, a spatially distributed network of stage gauges remains the best option to capture the observations required to feed the forecasting and data assimilation processes.

At the *Rambla del Albujón* watershed, we implemented a hydrological monitoring system consisting on a network of stage gauges located at eight critical junction points between major tributaries. The monitoring locations were carefully chosen in order to achieve effective water level monitoring during flood events and a reliable model-based forecasting system. Figure 1 shows the selected locations which are far away (∼50 km) from the control center at the University of Murcia, to the North of the watershed. In this area, an existing phone infrastructure enables the communication among the server in the control center and the DatalogV1s in the field. The DatalogV1s must be autonomous only with batteries, because no power source exists in the monitoring area and solar panels are frequently stolen or vandalized. In the following sections, we describe the design and development of the DatalogV1 to provide remote data gathering of the water stage in channels during floods.

## 4. Design of DatalogV1 Hardware

**Figure 2.**
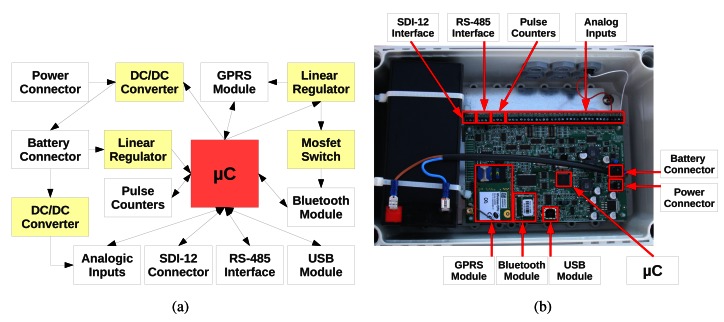
Two different views of the DatalogV1. (a) Block diagram showing the main components. (b) The electronic components and the battery are mounted on a IP65 protection box.

The DatalogV1's design was developed to address the requirements of the described application. The block diagram of DatalogV1 is illustrated in Figure 2(a). The critical components are a low-power microcontroller (*μ*C) module that supervises the DatalogV1's operation, multiple sensor interfaces (Pulse, SDI-12, RS-485, Analog) that enable to take measurements from different kinds of sensor devices, and a GPRS module for long-distance communication with the control center. Moreover, two communication modules (USB and Bluetooth) enable the in-situ interactions via a laptop. All electronic components and a battery are mounted in an IP65 waterproof box to protect from harsh weather conditions, as shown by Figure 2(b). The DatalogV1's design is balanced between low-power consumption for long-lifetime, and computational capability for multi-sensor reading and long-range communication. The hardware design of these components is described in the next subsections.

### 4.1. Design of Microcontroller Module

**Figure 3.**
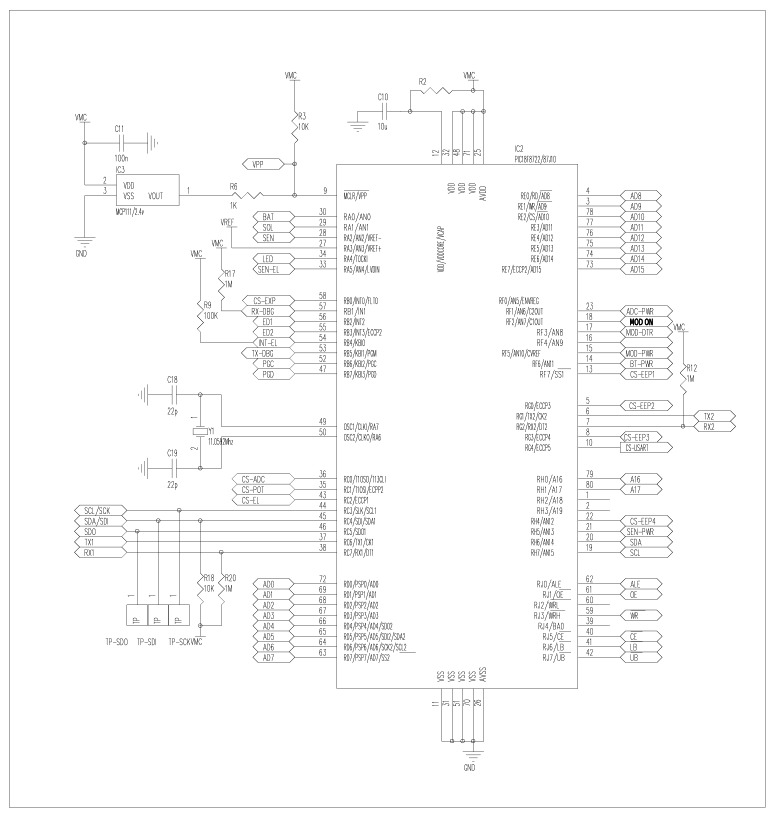
Circuit schematic of the microcontroller module. The center portion is the microcontroller used to control DatalogV1 operation, and the center-left is the crystal oscillator used for setting the clock.

The circuit schematic of the microcontroller module is shown in Figure 3. The central part of the schematic represents the low-power 8-bits microcontroller (PIC18LF8722) manufactured by Microchip. The PIC18F8722 operating to 3.3 V is ideal for low power applications (≃nanoWatts) with 120 *nW* sleep mode and 25 *μ*W active mode. It provides high processing speed (40 MHz) with a large 256 KB RAM memory. A 12 MB dataflash memory is included for local storage of sensor data. The top-left portion of the schematic (IC3) shows a security mechanism to avoid microcontroller blockage in case that available energy is not enough. Thus the microcontroller resets when there is less than 2.4 V. The center-left part of the schematic contains the crystal oscillator setting to 11 MHz. (OSC1/OSC2 tags). The oscillator provides a precise clock signal to stabilize frequencies for sensor readings and data transmissions.

### 4.2. Design of Sensor Interfaces

DatalogV1 provides multi-sensor interfaces to take readings from a wide set of hydrologic instruments. Its sensor interfaces are two pulse counters, two digital connectors (RS-485 and SDI-12), and eight analog inputs. Each pulse counter reads from a tipping-bucket rain gauge (pluviometer) which generates a discrete electrical signal for every amount of accumulated rainfall. Digital interfaces supply power to and read measurements from instruments, which can themselves include some degree of computational capability. Analog connectors enable the reading of simple instruments which modify the supplying voltages to return voltage values proportional to the physical observed variables. These multiple interfaces are compatible with the most of hydrological sensor devices in the market.

Pulse-counters typically connect to rain-gauge devices. The standard rain gauge collects the precipitation into a small container. Every time the container is filled and emptied, it generates a electric pulse. According to the number of pulses and the size of the container, DatalogV1 estimates the precipitation without requiring power supply.

For each digital interface, DatalogV1 can supply and read multiple sensors. Both RS-485 and SDI-12 interfaces consist of three electronic wires for data, ground and supplying voltage. The RS-485 is a standard serial communication for long distance and noisy environments. In addition, the SDI-12 is a serial data interface at 1,200 baud designed for low-power sensors. Using serial protocols, DatalogV1 can directly obtain the physical measurements.

The analog inputs allow to read 8 differential sensors, 16 single-ended sensors, or a combination of both options. A differential connection comprises four electronic wires acting as voltage-supplier, ground, positive-voltage, and negative-voltage, while a single-end connection contains two electronic wires for supplying-voltage and positive-voltage. The main difference between differential and single-ended is the way to obtain the voltage value. In single-ended, the voltage value is the difference between the positive voltage and the ground at 0 V. However, single-ended connections are sensitive to electrical noise errors, which are solved by differential connections. Because twisting wires together will ensure that any noise picked up will be the same for each wire, the voltage value in differential inputs is the difference between the positive and negative voltages.

**Figure 4.**
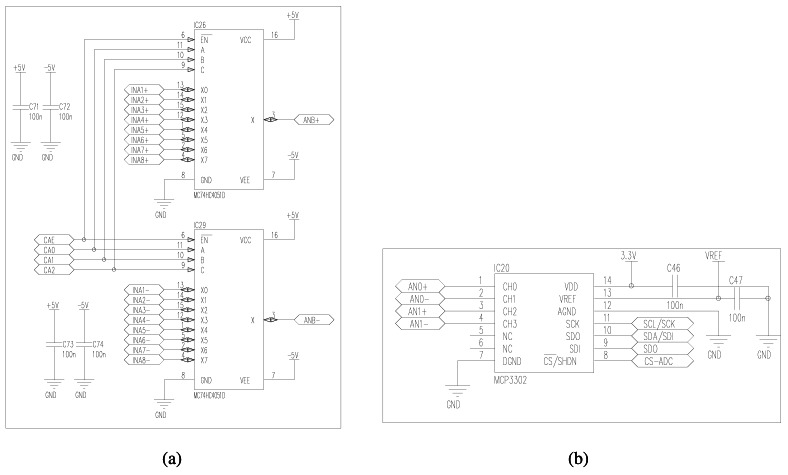
Circuit schematic of analog interfaces. (**a**) Selector of analog connections to plugged-in sensors, (**b**) ADC converter from output voltage to digital data.

To obtain the measurements of the physical variables, output voltages are processed using three main hardware components: multiplexer, amplifier, and ADC converter. Two multiplexers MC74HC4051D from Motorola company enable to select the output voltage of a specific analog sensor (Figure 4(a)). Each multiplexer contains 3 control pins CA0, CA1, and CA2 to choose an output voltage among 16 possibilities. The selected output voltage is amplified for preserving high effective resolution. DatalogV1 uses an AD8622 amplifier, manufactured by Analog Devices, that provides high current precision, low noise, and low power operation. The pre-configured amplification depends on the output range of the selected sensor. Finally, the amplified output signal is converted to a digital value through an Analog-Digital Converter (ADC), as shown by Figure 4(b). DatalogV1 contains a 13-bit ADC MCP3302, manufactured by Microchip, that provides high precision and resolution. This flexible design provides full compatibility with presumably all kind of available sensors for hydrologic use.

### 4.3. Design of GPRS Communication Module

**Figure 5.**
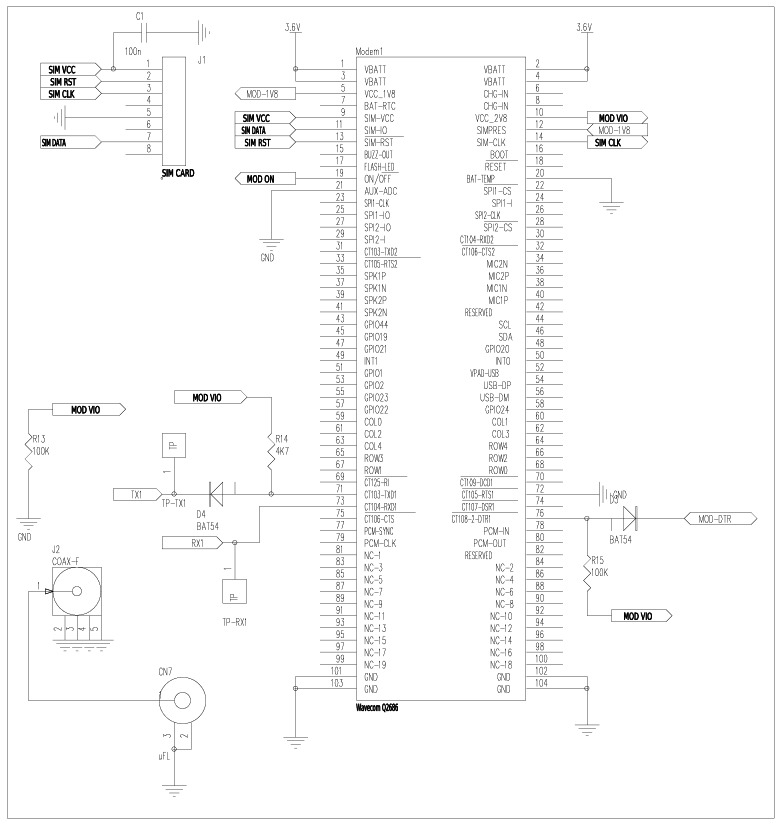
Circuit schematic of the GPRS module. The center portion is the GPRS module used to control the long-distance communication, and the top-left portion is the SIM card connection.

A GPRS module is used to transmit monitoring data from DatalogV1 to the control center. Figure 5 shows the GPRS module implementing all functions for wireless communications.

The top-left part of the circuit shows the connection of SIM phone-cards according to the manufacturer specification. The bottom-left shows a uFL coaxial connector to the wireless antenna. We chose a Wavecom Q2686 chip, which is connected to the microcontroller via an USART interface (CS-USART). The Wavecom Q2686 contains a programmable 256 KB SRAM memory and includes a ARM9 32-bit processor at 104 MHz. This Q2686 chip makes possible to join a GSM/GPRS base-station and receive/send data reliably in quad-band communications on the 800, 900, 1,800 and 1,900 MHz bands. Also, the chip makes it easy to upgrade to 3G when needed. This GPRS module enables long-distance UDP/IP communications through cellular radio networks.

### 4.4. Design of Power Module

**Figure 6.**
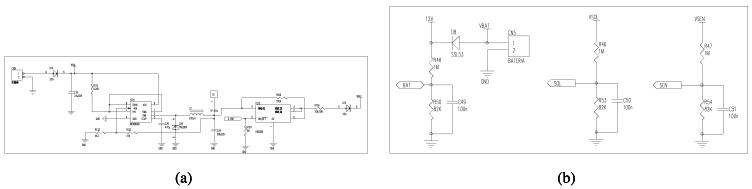
Circuit schematic of the battery, solar-panel, and power-control modules. (**a**) Battery and solar modules, (**b**) secure power control for battery, solar panel, and sensor.

The power module consists of two power sources and three regulable mechanism to provide a secure supply of electronics components. The main energy source is a 12 V DC battery of 7,000 mAh power capacity which can be rechargeable using an optional solar panel. To adapt the input tension of the solar panel (17–20 V) to a lower tension (12–15 V) to supply the battery, we use a commutated DC/DC regulator in step-down mode, as shown by Figure 6(a). The microcontroller turns on the DC/DC regulator when it detects that the battery has a low level according to a pre-established threshold. Three circuits guarantee stable energy levels for battery, solar-panel, and sensors, as shown by Figure 6(b). The circuits of battery and solar-panel include security mechanisms to avoid a too low power level input to the sensors. For this, the circuit of sensors is used, before readings are taken, to check if the power supply is stable as to obtain an accurate measurement.

**Figure 7.**
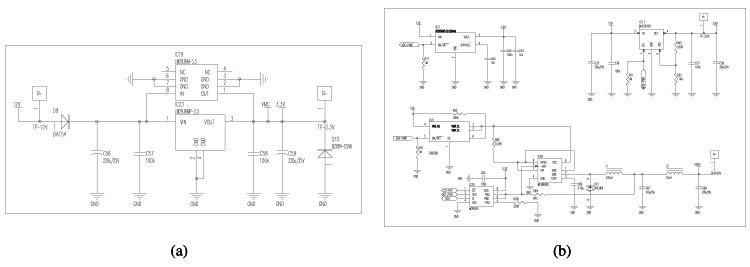
Circuit schematic of the power supply module. (**a**) Power supply for GPRS, sensors, and ADC converter, (**b**) power supply for microcontroller.

To reduce the power consumption, DatalogV1 keeps almost all electrical components deactivated, such as GPRS, sensors, and ADC. Only the microcontroller circuit is always supplied at 3.3 V (Figure 7(a)) through a linear regulator LM2936 from National Semiconductor with ultra-low current in the stand-by mode. This LM2936 regulator features low drop-out voltage (50 mA) to minimize power losses. Also, this circuit includes a diode (D10) to provide a security power to protect the microcontroller and all board at most 5 V. When it is necessary, the microcontroller supplies independently the electrical components using two DC/DC converters, two linear regulators and a MOSFET switch (Figure 7(b)). Concretely to supply sensors, a DC/DC converter and the MOSFET switch is combined to create a adjustable commutation cell. The design of the commutation cell includes high-power isolated chips in order to reduce interferences. At the same time, it has a good linearity and load regulation characteristics, and allows to establish the voltage supply between 3 V and 10 V. The chosen MOSFET is a FDC6330L, manufactured by Fairchild Semiconductor, which provides high performance for extremely low on-resistance (<0.2 V). The DC/DC used is a MC34063AD, manufactured by Texas Instruments, which make possible high voltage transmission. This technique provides a high power efficiency.

## 5. Design of DatalogV1 Software

This section explains the software design of DatalogV1 for semiarid hydrological scenarios. In semiarid zones, channels are often ephemeral, *i.e.*, they are often dry throughout the year as the result of infrequent and irregular precipitations, and flows can sharply increase during short storm events. Therefore, regular periodic monitoring provides little relevant information. Also, sensor acquisition and massive data transmission in dry periods leads to a waste of energy. Thus, DatalogV1 has been enabled to react to the rainfall-runoff events, providing only relevant data when needed and so reducing power consumption.

To address this issue, a low-consumption dynamic schedule is proposed for the major operations: sensor collection and data communication. This schedule keeps the DatalogV1 in sleep mode with all electrical components deactivated for most of the time. During the collection, the DatalogV1 is activated to supply sensor devices and take readings and afterwards it returns to sleep mode. The dynamic measuring schedule uses two different observation frequencies: low and high. The energy-saving low frequency is used most of the time in semiarid areas, as channels are commonly dry. When a relevant event is detected, the high observation frequency is activated. Also, only when a number of stored sensor measurements is reached, the DatalogV1 activates the GPRS modem to transmit data to the control server. This schedule takes useful measurements increasing the autonomy of the monitoring system.

The development environment for the DatalogV1 software is IAR Embedded Workbench for MicroChip, and the programming language is C [26]. The DatalogV1's software is divided into three main modules. First, the main loop module is responsible for initializing the configuration parameters and managing the low-consumption dynamic schedule for sensor reading and wireless communication. Second, the sensor reading program is in charge of the power supply to sensors and collection of sensor observations. Third, the wireless communication program is designed for receiving and sending data packets over a GPRS network based on UDP/IP protocol.

### 5.1. Main Loop Routine

**Figure 8.**
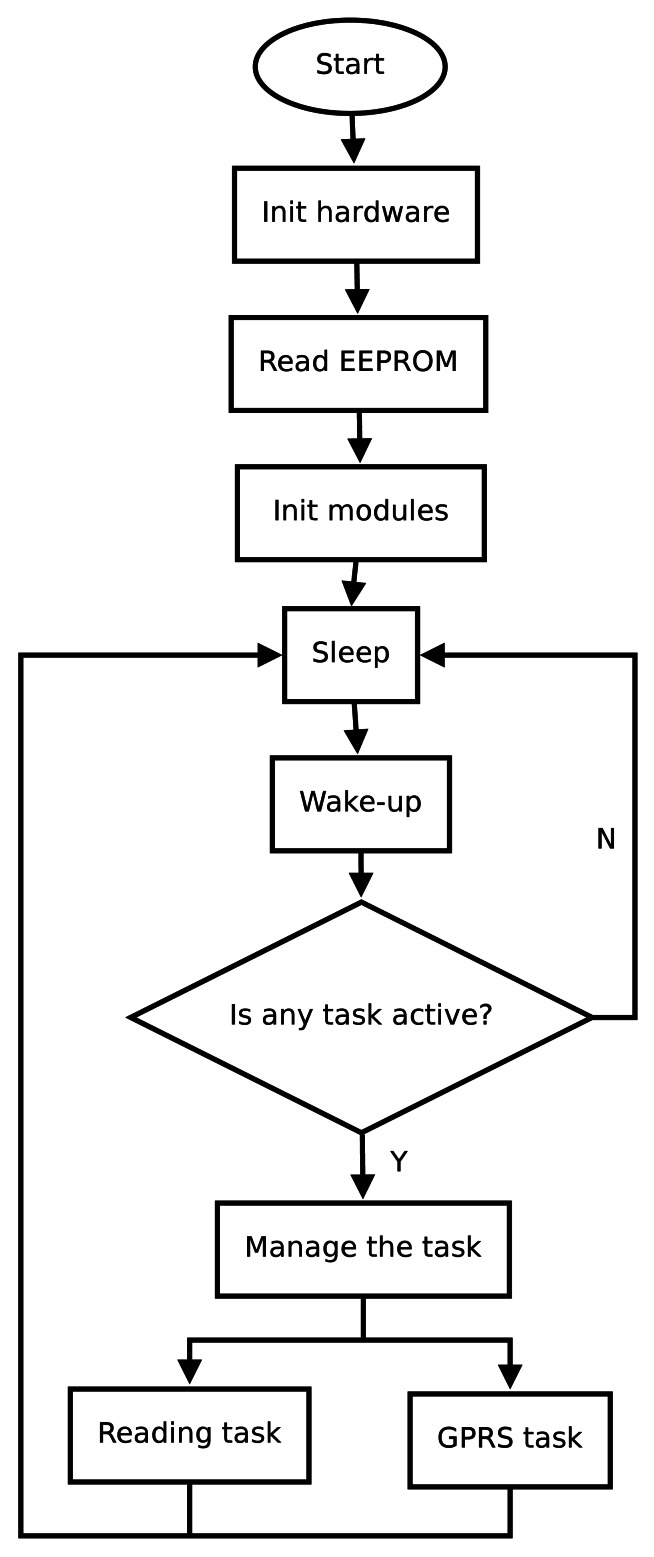
Flowchart showing the main loop routine of node operation.

The main loop routine performed by the microcontroller is illustrated in the flowchart of Figure 8. The operation of the main loop program is divided into six parts. First, the microcontroller initializes RTC clock, I/O ports, peripheral modules, and hardware interruptions. Second, the microcontroller reads EEPROM and saves in memory the configuration parameters of all operations. Third, the microcontroller configures the external modules, such as the GPRS module and sensors interfaces. Fourth, the microcontroller is kept in sleep mode during the most time possible and a watchdog process generates a wake-up interrupt every second. When the watchdog finishes the microcontroller resumes to working mode. Then, the microcontroller checks if there is some pending task to be done. Otherwise, the microcontroller must return to sleep mode. To indicate a pending task, the microcontroller uses flags in active mode. After dealing with the active tasks, the microcontroller returns to sleep mode.

The low-consumption schedule manages the activation of reading and communication tasks. The implementation of the schedule consists of a reading timer, a counter of stored sensor data, and a GPRS timer. By default, the reading timer is configured in low observation frequency, set to a measurement every 10 minutes. If the reading of a sensor exceeds a pre-established threshold, then a relevant event is assumed to happen, and the fast mode is activated, so the reading timer fires every minute. This way, the sensor data are saved until there is a pre-configured amount to transmit over GPRS network. In addition, a GPRS timer is used to guarantee a maximum time among transmissions, currently set to one hour. This schedule reduces the power consumption in reading and transmission, providing a reliable keep-alive system.

### 5.2. Sensor Reading Routine

**Figure 9.**
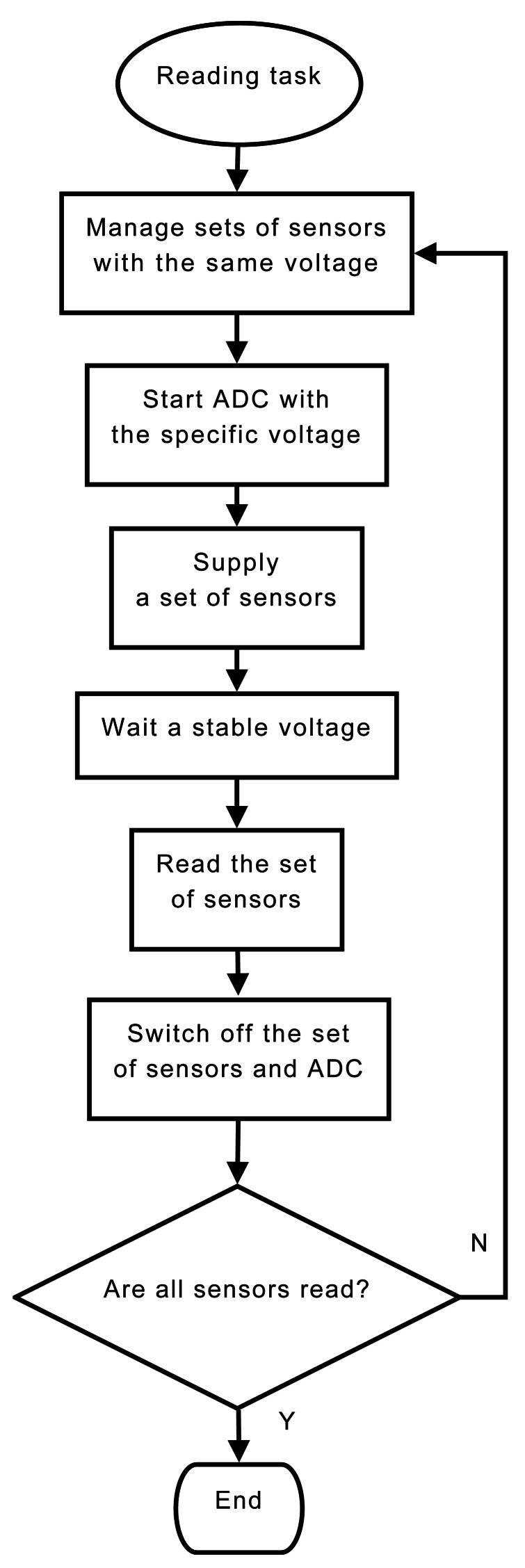
Flowchart showing the periodic task of reading several sensor with different power supply.

The reading routine must manage a wide range of hydrological sensor with different output types: pulse-counter, digital, and analog. The reading of a pulse-counter rain-gauge is based on a wake-up interruption, When rainfall accumulates, the rain-gauge generates an electrical signal which wakes up the microcontroller to keep a register of the number of pulses per hour. The reading of each digital sensor (RS-485, SDI-12) requires the use of a serial protocol to communicate with the sensor device. So, the microcontroller supplies the sensor devices during the measurement time and request the sensor for the physical parameter. The reading of analog sensors (differential, single-end) needs the use of an analog-digital converter (ADC) to obtain the physical measurements. Moreover, we group the analog reading of several sensors with common features in order to save supplying energy. Below, we explain in detail the reading process for analog devices according the flowchart of Figure 9.

In analog readings, the microcontroller divides all active sensor inputs into several sets with the same supplying voltage and the same output voltage. So, each sensors set is measured simultaneously. First, the microcontroller selects a set of sensor inputs and activates the ADC converter. The sensor devices and the ADC converter is supplied with the specific voltage. Then, the microcontroller waits until the output voltages are stable which is determined for the maximum measurement time of sensors selected. When the measurement time finishes, the ADC converts each read voltage output to the corresponding physical value. So, the microcontroller stores the measured values along with the time sequence when they were collected, and it switches off the sensors and the ADC converter. This scheme is repeated while there is some analog sensors which are still not reading.

### 5.3. GPRS Communication Routine

The main operation of GPRS communication is to transmit sensor data collected by the DatalogV1. In addition, the GPRS communication is in charge of sending health-status information and receiving software changes from the control server. To communicate with the control server, the DatalogV1 activates the GPRS module briefly, and then returns to sleep mode. The DatalogV1 and the control server follows the UDP/IP protocol, as it has lower complexity and consumption than TCP/IP.

**Figure 10.**
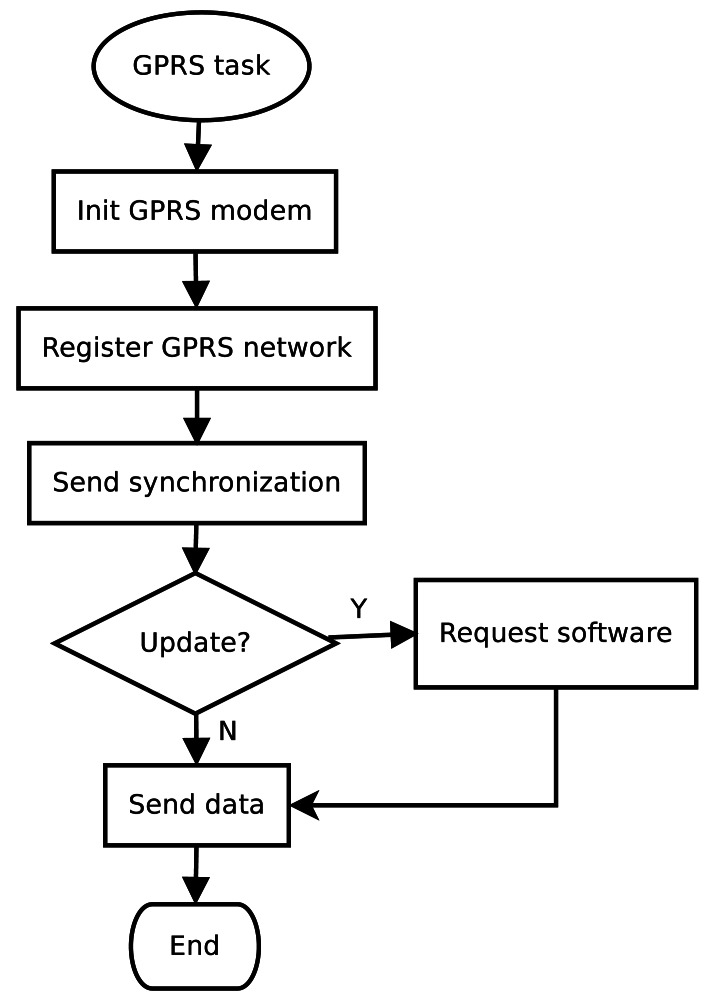
Flowchart showing the periodic task of GPRS communication from the node to the control server.

Figure 10 shows the flowchart of the communication routine divided into five parts. When the GPRS task is activated, the microcontroller switches on the GPRS modem, and sends a command with the configuration parameters of the communication. Then, the GPRS modem finds the nearest base station in its coverage area and establishes the connection conditions of the phone network. Once the GPRS modem is registered in the base station, the DatalogV1 sends a synchronization packet to the control server and waits the respective response. The response packet indicates if a new version of firmware or configuration is available. In this case, the DatalogV1 sends a software request and changes to the update state. During the update process, the DatalogV1 receives the firmware and configuration packets and commits the changes to itself. After the DatalogV1 is updated, the data sending is conducting. During the data transmission, the DatalogV1 sends sensor historical and health status packets to the control server. When the transmission finishes, the microcontroller switches off the GPRS modem.

Wireless communication is prone to transmission errors and intrusive attackers. To guarantee reliable communications, DatalogV1 provides three level of security: integrity, confidentiality and authentication. To implement those, we use three common mechanisms: CRC checking, AES/CBC encryption, and SHA-2 hash.

- CRC (Cyclic Redundancy Check) mechanism checks the packet integrity and detects error bits produced by wireless interferences.
- AES (Advanced Encryption Standard) in CBC mode is a symmetric-key encryption algorithm to provide a confidential communication. The AES function converts the input plaintext into the final output of ciphertext. To avoid the same ciphertext, we include a padding of random values.
- SHA-2 (Secure Hash Algorithm) implements a cryptography function to provide authentication purposes.

In addition, phone network limits the maximum transmission unit MTU to less than 2 KB. For this reason, the DatalogV1 must divide historical data into several frames ordering for time collection sequences. When the control server receives the data frame, it responds with an acknowledgement indicating the last sequence received. In the case of firmware of configuration changes, the control server divides the software update into several portions using offset sequences. When the DatalogV1 receives update frames, then it replies an acknowledgement response, which includes the last offset sequence of software received. This ACK scheme guarantees the bi-directional communication.

**Figure 11.**
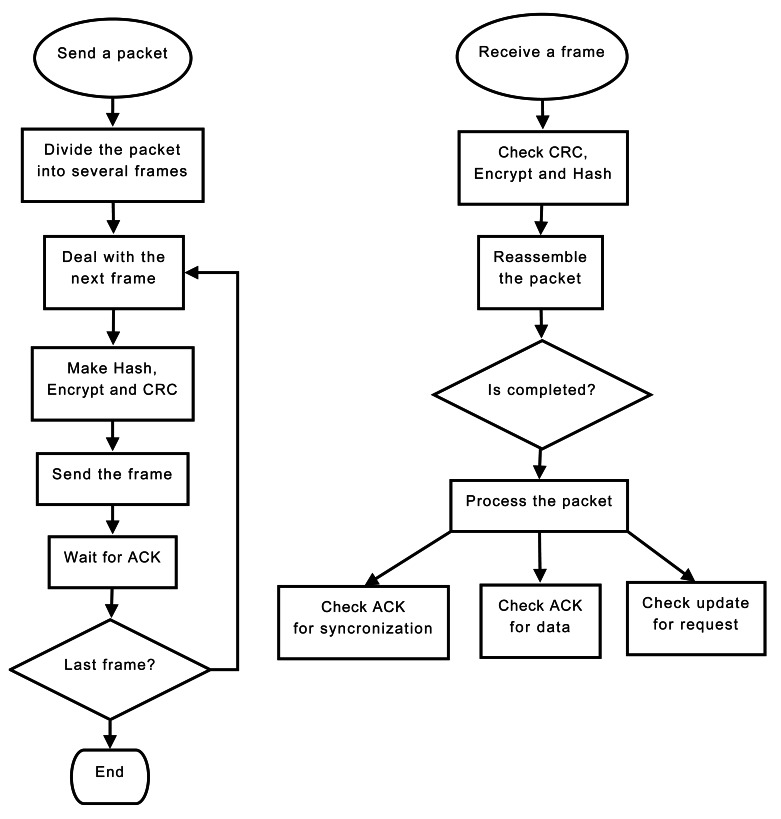
Flowchart showing the required task to send and receive frames using GPRS module.

**Figure 12.**
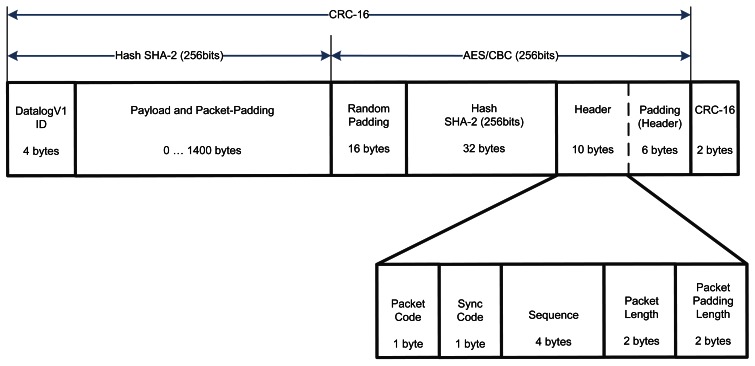
Depiction of the fields of the frame format used in GPRS communication.

To process frames, the microcontroller performs the sending and reception subroutines shown in Figure 11. In the sending subroutine, the microcontroller divides the GPRS packet into multiple frames. Each frame consists of a header, the identify of the DatalogV1, and the payload. A SHA-2 hash is applied to the payload and the identify to generate a 256 bits hash field. The header including the hash field is encrypted to generate a 256 bits unreadable header. For the payload and the unreadable header, the microcontroller determines the CRC value saved at the end of the frame. The created frame is sent and a timer is configured to wait the reception of its acknowledgement response. When a frame is to be received, the microcontroller should decide if all security fields are correct. So, the microcontroller checks the CRC field, decrypts the header and verifies the SH2-2 hash. If some checking is incorrect, the frame is discarded. In other case, the microcontroller extract the payload to reassemble the original packet. When the packet is received completely, it is processed. Figure 12 shows the format of the data frame used during the GPRS communication.

Harsh weather conditions may cause that DatalogV1 to have poor radio connectivity or hardware failures. To avoid information losses, the DatalogV1 provides a backup system based on persistent memory, cycle buffer, and keep-alive timer. The persistent 12 MB data-flash enables to store hundreds of thousands data registers during several days. The DatalogV1 implements a cycle buffer with two pointers that mark the last transmitted register and the last measured register. A keep-alive timer indicates the maximum response time from the control server to detect communication failures. When the timer finishes, the DatalogV1, previously to stop the communication, marks the last transmitted register that has been confirmed from the control server. The next occasion the GPRS communication is reestablished, the DatalogV1 transmits data from the transmitted register. This implementation provides a reliable communication solution to prevent information losses.

## 6. Remote Monitoring System

The monitoring system enables to gather sensor information from remote DatalogV1s and supports data analysis and decision-making. To administrate the monitoring system, a complete end-user interface carries out historical data queries and warning messages for health status. In hydrologic applications, the monitoring system enables the establishment of water level-based real-time alarm mechanisms for flood emergencies.

To provide data collection and storage management, we use a dedicated server running a Supervisory Control and Data Acquisition System (SCADA). The SCADA server is completely developed in JAVA Language. The SCADA implements a Programmable Logic Controller (PLC) to manage GPRS communication with each individual DatalogV1. Each PLC is charge for receiving sensor data and health status and transmitting software updates. To store all received information, the SCADA uses an object-relational database management system (ORDBMS), which currently is PostgreSQL v8.4. PostgreSQL is a free solution that supports almost all SQL constructs, transactions, and user-defined types.

**Figure 13.**
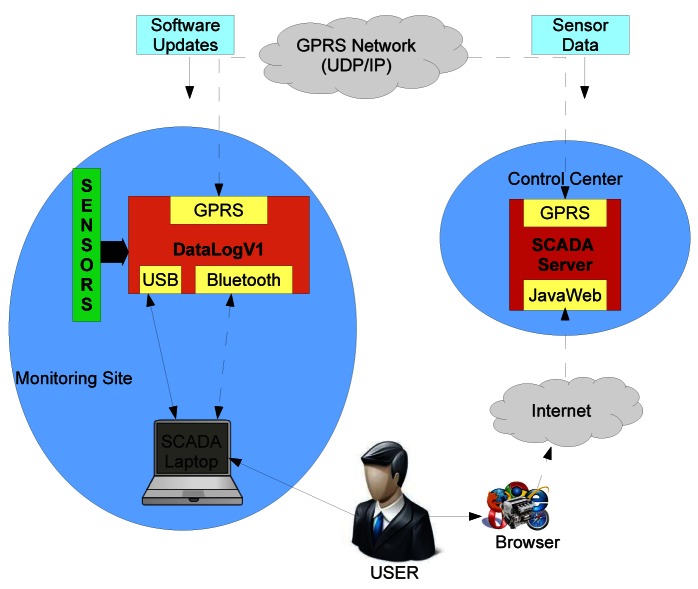
Monitoring system based on remote DatalogV1 and SCADA server.

To minimize the perturbation in areas of environmental interest, the monitoring system must allow remote maintenance tasks. In these cases, the objective goes toward zero human presence for maintenance and administration during the monitoring period. For this reason, the SCADA server provides a Java Web Start interface that enables the access to the monitoring system directly from the Internet using a standard web browser. Using the web interface, end-users can perform administration tasks, such as configuration changes, software updates, and health-status control. Also, these administration tasks are required during in-situ installation and removal situations. To assist in-situ interactions, a version of SCADA software for laptop has been developed. A laptop with SCADA can be connected to the DatalogV1 through both an USB connector and wireless Bluetooth communication. Thus remote and in-situ administration of the monitoring system is made possible. The proposed wireless monitoring system is shown in Figure 13.

The monitoring system provides data analysis for alarm messages by the web interface. The SCADA server analyzes and processes the water level which serve to (a) directly detect flood situations when the gathered data go up established thresholds, and (b) feed the model-based flood forecasting and data assimilation system. Issues of warnings will eventually be obtained for SCADA operators based on the two approaches, so the SCADA operators can more soundly decide on warning issues to be submitted to stakeholders. Currently, the first approach is fully implemented. The model-based flood forecasting system is in implementation phase. The approach is to assimilate flow depth data into a distributed hydrological model with semi-distributed parametrization to allow for model structure and parameter identification. The currently selected model is MARIAM [2]. The selected data assimilation method is the Monte Carlo based Ensemble Kalman Filter (EnKF) [27], which even under Gaussian assumptions, allows for some non-linearities to be updated in the ensemble during the assimilation step. We are currently evaluating several available options and variations of the original filter. In addition, the SCADA checks the health status of DatalogV1 to enable warning messages when the communication period or low battery deviate from normal situations. Its functionality provides reliability data gathering and fine-grained control without human presence in the field.

## 7. Deployment of Monitoring System: Hydrologic Sensor and Protective Structure

Selection of hydrological instrumentation is an important factor in the autonomy of the monitoring system, as reading of sensor measurements is a frequent and high power consuming task. This consumption not only depends on the nominal power required by the sensor but also on the time it requires to take a reading, including excitation time. For our application, we used 7 pressure sensors (UNIK-5000; *General Electric Sensing*), which provide high accuracy and fast measurement time, leading to low power consumption. UNIK-5000 sensor has a piezo-resistive pressure transducer, which measures the water pressure at the end of the sensor probe and returns a voltage output in milliamperes (4–20 mA). The vertical accuracy of the sensor is 10% of the measurement range, which in our deployment was 0–5 m or 0–7.5 m, depending of the depth of the channel cross-sections and maximum expected flow depths. DatalogV1 maps the voltage readings into the water column (cm) by using a tank-calibrated linear function.

To map water columns to flows, a channel recognition and GPS/GLONASS field campaign was conducted obtaining the definition of channel characteristics and slopes. At every measurement location, we took a 3 cross-sections as far apart as possible (order of several hundred meters) within channel transects which we considered relatively uniform (so changes in flow regime are highly unlikely in the transect). That is, one at the measurement point, another upstream and yet another downstream. The location of the water depth probes was precisely measured within each central cross-section. Rating curves to map water levels to flows were obtained through simulating steady-state conditions and subcritical flow with the hydraulic simulation software HEC-RAS [28], and applying a Manning's friction coefficient *n* = 0.035.

**Figure 14.**
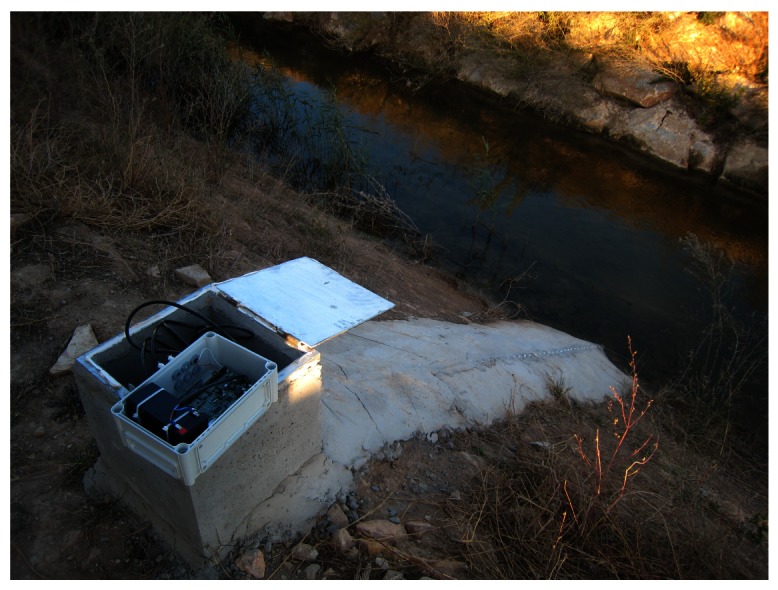
Protective deployment in the Albujón watershed. The DatalogV1 device is inserted into a 400 × 400 cm cement housing. The housing has 70 cm diameter hole that connects a 65 cm diameter and 7 m length PVC pipe, which protects the UNIK-5000 sensor.

Debris is common in channelized flow in flash floods all over the region. Thus, we used a protective safety structure for the UNIK-5000 sensors, as shown in Figure 14. That is, the UNIK-5000 sensor is placed inside a PVC tube, which has an array of small orifices along its axis. Orifices are transversal to flow, so water pressure is hydrostatic, and the uncertainty in water level measurements due to inertial energy is negligible. The UNIK-5000 is wired to the DatalogV1 located at the top of the PVC tube. The design allows water entering into the pipe, while preventing debris impacts.

Within the *Rambla del Albujón* deployment, a special measuring location is a city center where vandalism is likely To confront this problem, we selected a Radar Level Sensor (OTT RLS), which enables accurate and high resolution measurements (cm) without physical contact with the water flow. The OTT RLS sensor has a very high consumption. Fortunately, we could plugged into the public electricity grid to provide supply for the DatalogV1 device at this specific point. Both the DatalogV1 and the OTT RLS sensor were perched below a high-level bridge (10 m height) to prevent vandalism. Table 1 summarizes the features of the deployed hydrologic sensors.

In additional, rainfall measurements are available from a existing dense rain gauge network deployed in the *Rambla del Albujón* watershed. The network comprises two subnetworks, both of them with tipping-bucket gauges with a resolution of 0.2 mm. One is operated by the Agroclimatic Information Service of Murcia (SIAM), and the other belongs to the Automatic Hydrological Information System (SAIH), operated by the Water Authority (Segura Basin Hydrological Confederation; CHS). The spatial coverage is fairly uniform, and 16 rain gauges fall within the watershed or in a near neighborhood. Data are also transmitted in real-time from these collaborating organisms to the SCADA system.

## 8. Testing Results

The goal of our field development is to provide a long life-time monitoring system to obtain continuous accurate water level information to be assimilated into a model-based flood warning system. Here we focus on two tests conducted to assess the success of the monitoring system. The first test was done to validate the functional operation in terms of robustness and reliability. The second one was performed in order to assure the low-consumption and the autonomy during a complete hydrological cycle.

### 8.1. Hydrological Monitoring Results

**Figure 15.**
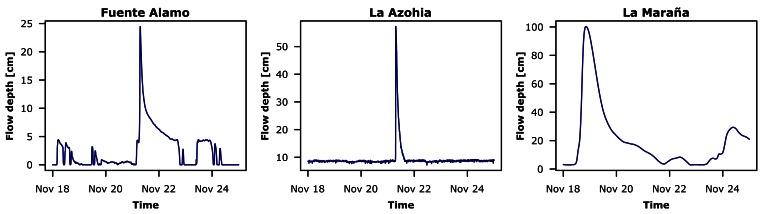
Water level collected during one week, corresponding to a storm event in November 2011. The plots show the gauges which recorded a significant flow during the storm event.

The deployment to test the functional operation was done in the *Rambla del Albujón* watershed, on the 28 October 2010. Usually, the spatial variability in semiarid hydrological systems is high. Thus some nodes may well be recording flow data while other stay dry in other watershed areas. Up to date, just some of the DatalogV1s wired to UNIK-5000 sensors have captured relevant information. This is not hydrologically uncommon, due to the general lack of precipitation in the given semiarid environment, where flash floods are occasional but occur abruptly every few years. Still, the available measurements serve well to the purpose of evaluating the monitoring system. The most significant event since the deployment occurred during one week in November 2011. Figure 15 shows the water level readings gathered by the gauge stations which recorded significant data. Despite existing harsh weather conditions, the monitoring system showed it robustness and reliability, communicating the collected measurements to the central base without any data losses.

### 8.2. Autonomy Results

The DatalogV1 was designed and developed for energy efficiency. The consumption depends on the pre-established configuration. In the worst case (high observation frequency mode), the DatalogV1 runs a data reading every minute and a data transmission every hour. Using a Tektronic TDS 3014B oscilloscope, we measured the power used for the main periodic states: sleep, sensor acquisition, and GPRS transmission. In easier terms, GPRS transmission is divided into two subtasks: registration and communication. Table 2 shows the approximate power and duration of each task. The average consumption *E* per hour is denoted as:

(1) $$E_{\text{state}}=\text{Power}\cdot \text{Duration}/\text{Frequency}[mAH]$$

(2) $$E=E_{\text{sleep}}+E_{\text{acqui}}+E_{\text{regis}}+E_{\text{transs}}=0.352mAh$$

where Equation (1) estimates the power used for each state and Equation (2) indicates the total consumption, which is 0.352 mAh. As expected, the GPRS module consumes most of the energy while the chosen UNIK sensor and microcontroller are very power efficient. Using a 7,500 mAh battery, the autonomy is 887 days, which is more than enough to monitor one annual hydrological cycle.

## 9. Conclusions and Future Works

The paper describes the design and implementation of a real-time monitoring system for hydrologic applications. The proposed monitoring system presents useful characteristics as large network capacity, sensor hardware compatibility, low power consumption, low cost, and minor impact on the natural environment. The testing results shows that the system is energy efficient, has a robust communication capability, and provides real-time accurate measurements. Its flexibility enables a wide application span for autonomous data collection with reliable transmission in few sparse points over large areas. We are currently evaluating the incorporation of a ZigBee radio module in the design to be able to gather data from wireless sensor networks in zones with dense measuring points. Also, hydrology-related current work involves the real-time assimilation of gathered data into a model-based forecasting system.

## Figures and Tables

**Table 1.** Features of hydrologic sensors deployed in the field.

Feature	OTT RLS	UNIK 5000
*Measuring Parameter*	*Water Level*	*Water Level*
*Interfaces*	*SDI* – 12	4–20*mA*
*Resolution*	0.35*m H2O*	0.5*m H2O*
*Vertical Accuracy*	±0.10%	±0.10%
*Measuring Time*	20 *sec*	0.01 *sec*
*Power Supply*	9 – 28 *V DC*	7 – 32*V DC*

**Table 2.** Power consumption and duration of each periodical state.

State	Power (mA)	Duration (sec)	Frequency (sec/hour)	Consumption (mAh)
*CPU Sleep*	0.095	3, 600	3, 600	0.095
*GPRS Transmission*	700	1	3, 600	0.194
*GPRS Registration*	50	3	3, 600	0.042
*Sensor Acquisition*	63	0.02	60	0.021
